# Implementation of an embedded behavioral health care model in a pediatric rheumatology subspecialty juvenile myositis clinic

**DOI:** 10.3389/fpsyt.2023.1192711

**Published:** 2023-08-10

**Authors:** Susan Shenoi, Suzanne E. Edison, Stacey Haynes, Joanna Patten

**Affiliations:** ^1^Seattle Children’s Hospital and Research Center, University of Washington, Seattle, WA, United States; ^2^Mental Health Coordinator, Cure JM Foundation, Leesburg, VA, United States

**Keywords:** mental health, pediatrics, juvenile myositis, behavioral health, rheumatology

## Abstract

Youth with chronic medical illness, such as juvenile myositis (JM), require specialized behavioral health care. However, access to such care is challenging due to the youth mental health crisis, which impacts accessibility of mental health services in the community, as well as challenges accessing behavioral health care above and beyond the demands of care related to their JM management. In this paper we describe an embedded behavioral health care model, including the establishment and implementation of such a model, at a pediatric hospital where youth with JM receive medical care in a Center of Excellence (CoE). We describe a unique partnership with a philanthropic organization; the challenges and benefits of delivering care within this model; as well as recommendations for maximizing its effectiveness. Ultimately, we provide an example of a successful embedded behavioral health care program for youth with rare disease, which may be applied to other institutions providing similar care.

## Introduction

Juvenile myositis (JM) is a rare, chronic autoimmune disease with an incidence of 2–4 per million children in the United States (1). The illness is characterized by inflammation in the blood vessels under the skin and in proximal muscles and requires long term immunosuppressive medications often including oral or intravenous steroids for disease control (2, 3). Youth with chronic rheumatologic or autoimmune diseases often have coexisting mental health symptoms, including depression and anxiety (4, 5). These co-occurring mental health symptoms are difficult to address, particularly in the context of the youth mental health crisis and associated mental health provider shortage, which has been declared a national emergency (6, 7). Multiple healthcare groups and the US Surgeon General have advocated for diversifying care delivery models to meet the growing youth mental health crisis (8). One such model is embedded behavioral health care (EBHC), which provides coordinated, early detection and intervention for behavioral health needs within primary or subspecialty pediatric medical settings. Youth with JM may access subspecialty pediatric care at a JM Center of Excellence (CoE) in the United States, where highly specialized rheumatological and other interdisciplinary care is available (9, 10), although to our knowledge, none of the five JM CoE’s include EBHC.

In this paper, we describe partnership between the Cure JM Foundation and the JM CoE at Seattle Children’s Hospital (SCH) in the development of an EBHC model to meet the behavioral health needs of youth with JM. We describe each component of the implementation of this model, including the process of establishing interdisciplinary alignment, obtaining strategic funding, and defining the scope of behavioral health care practice. Reflecting on our experience, we provide recommendations for EBHC that can be replicated at other JM CoE’s or in other pediatric subspecialty care settings.

## Context

### Pediatric rheumatology’s current state

Pediatric rheumatology is an emerging pediatric specialty, and many states lack rheumatology specialty care. The American College of Rheumatology Workforce study in 2020 estimated there were approximately 300 practicing pediatric rheumatologists in the United States as of 2015 and estimated that 95 additional pediatric rheumatologists would be required to meet demand, with projected worsening of pediatric rheumatology specialists to requiring roughly 230 pediatric rheumatology providers by 2030 (11). Youth with chronic illness, especially if symptomatic with active disease, are more likely to be followed in pediatric subspecialty settings, where overburdened pediatric rheumatologists may be focused specifically on JM illness management, at the potential risk of overlooking behavioral health problems that might otherwise be addressed in primary care settings.

### Embedded behavioral health care model current state

Pediatric rheumatology subspecialty clinics are an opportune health care contact point for youth with JM to receive targeted, preventative behavioral healthcare. Parents of youth with JM report difficulty accessing behavioral healthcare services in the community and endorse preference for embedded behavioral health screening in JM specialty care (12). Developing an EBHC model to include preventative screening in such a subspecialty care setting is aligned with key recommendations for youth behavioral health care (6). In the EBHC model the multidisciplinary team (rheumatology provider, behavioral health provider/therapist, social worker, other health care providers as needed) meet at a single time point with the patient and their family, thus providing co-located care at a medical visit to maximize access and improve outcomes.

### Philanthropic partnership

**Figure 1:**
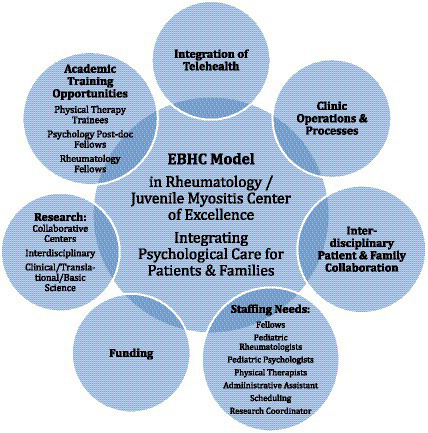
Different aspects of pediatric rheumatology subspecialty juvenile myositis (JM) clinic embedded behavioral health care (EBMC) model for mental health care. EBHC, embedded behavioral health care; PT, physical therapist; Ped, pediatric; Admin Asst, administrative assistant; JM, juvenile myositis.

The Cure JM Foundation, founded in 2003, is a nonprofit, organization whose board is led by families or friends of patients with JM (13). Cure JM Foundation’s mission is to find a cure and better treatments for JM and to improve the lives of families affected by JM. Mission-driven priorities include funding JM research, providing education to JM families and providers through symposiums and conferences, and supporting JM families and patients with workshops and support groups. Cure JM collaborates and builds strategic partnerships with key institutions to facilitate advocacy and research efforts to advance the understanding and treatment of JM. Cure JM has funded and helped establish five CoE in leading pediatric institutions across the United States. A priority of the foundation has been to increase access to behavioral health care services within the CoE to address the unique needs of patients with JM including hiring of a dedicated expert (SE co-author of this paper) as the mental health coordinator to promote this initiative (see Figure 1).

### Subpopulation behavioral health care needs

Youth with JM have unique health needs that may be associated with behavioral and emotional health challenges (Table 1). Chronic steroid medications can result in cushingoid appearance, which has been associated with low body-image and self-image in youth (14). Medication-related side effects as well as chronic symptoms secondary to JM (including but not limited to pain, fatigue, weakness) can impact youth’s ability to engage in physical, educational, social, and recreational activities. Due to risk of flare with exposure to ultraviolet light, youth with JM must avoid the sun, which can impact social and recreational opportunities. Medical care, including periodic hospital admissions, frequent clinic visits, frequent blood draws and need for long term oral as well as injectable medications, can place a high burden of disease management on youth with JM and their families. It can be challenging for youth to cope with the unpredictability of JM flares, the nature of a complex and rare disease, and lack of understanding from social supports (e.g., friends, school personnel, and extended family) about their illness and medical management needs. Additional challenges of this illness include lack of clarity in the rheumatology field around JM etiopathogenesis or triggers, lack of clinical trials documenting effectiveness or comparative effectiveness of medications, and varying treatment plans for management of JM mostly based around consensus or expert opinion, which leads to variability in provider recommendations for treatment.

**Table 1:** Challenges of juvenile myositis (JM) at the patient, caregiver, and system levels.

Challenges for caregivers	Challenges for patients	System level challenges
Long wait times for appointments Inconsistent insurance coverage Cost of medical care Cost of loss of work due to appointments, illness, flares Unpredictability of disease flares or medication effect and precision of therapy for individual patient Lack of understanding from friends and relatives about JM Emotional burden of managing the impact of JM on siblings or other extended family members	Inconsistent ability to participate in sports or social activities due to disease flares School absence for medical appointments, flares Medication related factors including side effects, long term need for subcutaneous or infusion medications Risk of flare with sun-exposure Risk of long term steroid medications, including but not limited to cushingoid appearance, osteoporosis, diabetes, hypertension, cataracts, growth failure, etc. Chronic discomfort of JM symptoms including pain, muscle weakness, and rash around face, arms, chest etc. Frequent blood draws for disease and or medication monitoring Feeling isolated due to feeling different from peers	Pediatric rheumatology and mental health workforce shortages Multidisciplinary clinic coordination challenges Lack of consensus on JM etiopathogenesis or triggers, leading to variable treatment regimens across JM patients Lack of clinical trials documenting effectiveness or comparative effectiveness of medications for JM Lack of interested stakeholders (pharmaceutical companies etc.) in supporting medication trials for rare diseases

Ardalan and colleagues assessed parental preferences and perspectives around the mental health needs of their children with JM using focus groups at the Cure JM Foundation national family conference. Results indicated intense patient and family distress associated with JM and emotional themes that emerged included parental concern for child depression and anxiety, parental sadness, grief, anger, guilt or anxiety, and family dynamic concerns for distress in siblings of youth with JM. Four themes emerged in the screening and interventions domain of their study including (a) patient transparency (patients having hesitancy to disclose true feelings/emotions), (b) barriers around emotional health resources, (c) provider role around facilitating emotional health, and (d) preferences around possible intervention modalities. Based on their findings, Ardalan et al. recommend routine behavioral and emotional health screening with patient self-report and parent-proxy report on validated measures to identify the behavioral health needs at pediatric rheumatology visits. The authors also recommend co-location of psychological services such as an EBHC model in pediatric rheumatology clinics using innovative psychosocial interventions and maximizing technological modalities with both online and in-person structured peer support (12). This study leveraged input from a large representative geographic sampling of families and patients with JM. Encouraging findings include perceived resilience in young adults with JM as well as high levels of trust, rapport, and comfort between youth with JM and their parents with their rheumatologists and/or primary care providers. These findings support an EBHC model in rheumatology subspecialty clinic to increase access to standardized behavioral health care for this vulnerable and unique population.

## Key programmatic elements

### Model of care

The Cure JM Foundation, in partnership with the established SCH JM CoE (established 2019), a quaternary care hospital in Seattle, WA, discussed various clinical models and funding sources for integrating behavioral health care into the rheumatology specialty clinic. The goal of integrating behavioral health services in the JM CoE was to provide mental health symptom screening, referral for treatment of the child’s mental health needs, and to provide behavioral health intervention at the intersection of child’s developmental trajectory due to impact of JM symptoms and/or treatments. Rather than focus our behavioral health services on diagnosing psychopathology (which is more readily available in community mental health settings), our model, which underpins principles of EBHC, makes use of the expertise of behavioral health providers who can conceptualize the impact of JM symptoms and treatments on the psychosocial ecosystem of a child or adolescent at different developmental stages over time. Behavioral health providers in JM can provide brief interventions to address psychosocial concerns that may not rise to the level of acuity to receive mental health treatment in the community but are deserving of clinical intervention and in an effort to prevent symptom exacerbation. Some of these concerns can include adjusting to a new diagnosis, managing impact of diagnosis or symptoms on social, academic, or recreational activities, adhering to medical recommendations or treatment plans, pain management, and self-management as adolescents transition to adult health care systems.

Screening for symptoms of anxiety and depression, both of which are more common in chronic disease rheumatologic states (5) are incorporated annually to maximize early intervention efforts. We selected brief patient self-report and parent-report screening tools for our clinic to include a wide age range of patients. The measures we chose include: The patient health questionnaire-9 (PHQ-9) (15), which is a non-proprietary patient self-report brief screen of depression symptoms in patients age 12 and older; the generalized anxiety disorder 7 (GAD-7) (16), which is a non-proprietary patient self-report brief screen of anxiety symptoms in youth 13 and older; and The pediatric symptom checklist (PSC) (17), which is a non-proprietary parent-report measure of a range of psychosocial problems in children ages 4 to 17 years. In addition to standardized mental health symptom screening, the psychologist also provides emotional support, and psychoeducation for parents and patients (Supplementary Table S1).

### Clinical operations and process of EBHC in SCH JM CoE

Clinical operations are discussed as they pertain to the following: (A) clinic establishment and flow, (B) integration of telehealth, training of fellows and research, (C) staffing requirements, (D) interdisciplinary and patient/family collaboration, and (E) funding.

(A) Clinic establishment and flow: the JM CoE clinic occurs two, half days per month during which JM patients are seen individually with their parent for an hour return or follow-up visit. This allows the CoE to provide continuity care for a revolving eight patients per month. Since its inception in 2019 the CoE has served 51 unique JM patients with 213 visits for these patients from August 2019 to December 2022. A needs assessment survey of JM CoE families was conducted by the rheumatology administrative staff (patient support coordinator) prior to initiation of mental health care in clinic. Families unanimously stated they preferred seeing a psychologist at their routinely scheduled CoE clinic visit. Thus, the JM CoE implemented an integrated EBHC model with a psychologist in its second year of establishment (2020). The psychologist is co-located in the JM clinic and is scheduled to see every patient following their joint rheumatology and physical therapy visit. All visits with psychologist (initial and return are scheduled for 60 min. If indicated the psychologist can provide additional individual behavioral health follow-up care outside of the JM clinic. An introductory letter was sent to the JM CoE families to provide information on the new model of care. Families were allowed to opt out of the psychologist visit at their discretion (thus far only one family has chosen to opt out from all visits with psychologist). A resource list of electronic mental health resources was created by Cure JM in 2022 for use to provide additional information to families (Supplementary Figure S1). The rheumatologist and psychologist met monthly to troubleshoot and debrief clinic flow and discuss any ongoing or new needs during the first and second year of the clinic establishment.

Although the added behavioral health specialty care results in a longer visit (by about 40–60 min) this integrated, interdisciplinary model reduces overall care burden on patients and families by consolidating scheduling, transportation, and appointments into one clinic visit. Rarely, if a behavioral health crisis is identified during screening, additional time is needed to complete additional safety assessment and planning. If the behavioral health provider was not available or present in clinic, a collaborative agreement with the Social Work department to respond to identified crises has been necessary. Families have also reported reduced disruption of work, school, and extra-curricular activities, decreased need to arrange multiple episodes of childcare for siblings, and less demand for additional planning overall. The JM CoE template was built as a multidisciplinary template in the electronic medical records (EMR) with a single comprehensive note for each visit that includes the rheumatologist, physical therapist, and psychologist’s documentation. Creating this multidisciplinary separate template in the EMR allowed visibility, access to records, single note charting/documentation, and allowed billing capture for all three providers. However, the rheumatologist, on whose template the patient is scheduled with, must sign off after all documentation is completed by each of the individual providers.

(B) Integration of telehealth, training of fellows, and research: the COVID-19 pandemic in March 2020 was unexpected and prompted incorporation of telehealth as part of the CoE. Multidisciplinary telehealth visits were conducted with the three CoE providers in different locations (typically their home or clinic offices) during the peak of the pandemic when social distancing was appropriate. This was done via EMR zoom telehealth links and was found to be seamless and an acceptable method of providing both rheumatologic and psychological care. Since ending of the COVID-19 pandemic families are seen in person but have been allowed to continue telehealth care on and as needed basis.

Integration of pediatric rheumatology fellows into the CoE was done at its inception with expectations of each fellow to attend two CoE clinics each year of fellowship for a minimum total of six clinics throughout their training. Although there are no formal expectations of fellows to observe or participate in behavioral care training at our institution, shadowing the psychologist was an optional training experience within the EBHC model. Additionally, this model has served as a resource for several physical therapy students and learners to observe and participate in learning around JM assessments.

Our CoE participates in field-advancing research, which often includes multisite studies that require regular collection of validated outcome measures for JM including disease activity and other laboratory measures for disease activity or quiescence. The presence of an established EBHC model facilitated participation in research studies that also included behavioral health components. The presence of a psychologist and rheumatologist at the same clinic visit streamlined recruitment, consenting, participation and engagement of families in JM related mental health research. The benefits of this can be far-reaching as they not only allow for excellent care but facilitate much needed progress in the research arena for these rare diseases. We were able to launch a collaborative research study enrolling patients in a short timeline as a site of care that assessed acceptability of mental health screening, rates of positive mental health screening results, and associations of mental health with outcomes/health behaviors in JM (18).

(C) Staffing requirements: comprehensive and adequate staffing for the EBHC model includes the following: (1) providers including (a) pediatric rheumatologist with interest in facilitating the above goals (ideally the CoE clinicians), (b) pediatric psychologist or other licensed behavioral health provider with interest and expertise in rheumatologic or autoimmune diseases, and (c) trained physical therapist with interest in myositis; (2) administrative staff including; (a) operations manager (or administrative assistant, provider deployment coordinator) in rheumatology and behavioral medicine that helps build templates, schedules and provides other administrative support such as follow up surveys etc., a qualitative follow up survey was conducted a year into the implementation of the model by administrative staff. Of 15 random COE families that were attempted to be reached, 8 responded and 7/8 noted they liked seeing the psychologist. One family thought a “psychologist was not necessary.” (b) Scheduling team staff to build EMR scheduling templates and contact families to schedule visits.
(D) Interdisciplinary and patient/family collaboration: interdisciplinary collaboration and alignment on the clinical priorities and operational deployment of the EBHC model are important to establish at the outset of developing a new program. It is also critical to center the perspectives and experiences of patients and families with JM in this alignment through needs assessment or follow up surveys or focus groups as key stakeholders. In our model, the Clinical Director of Rheumatology and the JM CoE and a parent advocate who served on the CoE’s Steering Committee and was a former board member of Cure JM Foundation approached the Chair of Psychiatry and Behavioral Medicine to express interest in deployment of behavioral health services in the JM CoE at the same institution. Once leadership aligned on the goal of increasing behavioral health services for the JM population, the service line lead for embedded behavioral health programs at the same institution became involved in discussing possible mechanisms to staff a pilot of this program.
(E) Funding: the time commitment proposed for embedded behavioral health presence was approximately 16 h per month which would allow for direct face to face time of 8 h/month during clinics and additional time outside of clinic for nonbillable clinical coordination, consultation, and additional patient follow-up needs. Initially there was no identified funding mechanism for this work. Due to funding constraints the first year of this model was piloted with a psychology postdoctoral fellow (0.1 FTE) who had flexibility in their time and identified training goals that aligned with program development and clinical practice within an EBHC model. The fellow was supervised by a licensed pediatric psychologist during the pilot phase. For the second year of this model a pediatric psychologist’s salary was funded (at 0.1 FTE) through a combination of institutional (SCH Foundation 50%) and private philanthropy (50%) funding. The Psychiatry and Behavioral Medicine Department was able to identify a pediatric psychologist with interest in chronic disease who was well suited to continue the EBHC in JM CoE.

## Discussion

Despite national healthcare recommendations and parent preferences for embedded behavioral health services for youth with JM, access to this model of care is a rare commodity. Knight and colleagues (2019) surveyed behavioral health care providers including social workers and psychologists at institutions that are Childhood Arthritis and Rheumatology Research Alliance (CARRA) sites and found that a third of sites (*n* = 100) did not have access to behavioral care. These behavioral health providers noted that mental health interventions are a significant unmet need for pediatric rheumatologic conditions. They identified gaps and needs in care in several areas including need for clear pathways or protocols for mental health screening, need for follow up plans for mental health care, need for interventions targeting disease related anxiety, adjustment, or coping, need for support during transition to adult care, and need for addressing parent and caregiver related stress (19). Although there has been increasing attention to the role of embedded behavioral health providers in pediatric primary care settings, the prevalence of embedded behavioral health services in pediatric specialty care varies substantially by pediatric institution, and is typically concentrated in larger illness groups (e.g., diabetes) (10, 20). Embedded behavioral health treatment models have been successful in increasing access to treatment for youth in primary care settings (21). However, models for establishing embedded behavioral health programs in rare pediatric specialty programs or emerging pediatric specialties such as pediatric rheumatology have not been empirically described. We describe a uniquely innovative model of embedded, co-located, and integrated behavioral and mental health care (EBHC) for a rare chronic pediatric disease population that we believe is the first of its kind.

Mental and behavioral health are common comorbidities of chronic pediatric disease (22), and our model is unique in its engagement of multiple stakeholders to develop, fund, and implement a preventative EBHC model within JM. In addition to the benefits that this model provides to patients and families, the EBHC model allows pediatric rheumatology providers to focus on rheumatological disease aspects during their visit while leveraging the expertise of the behavioral health care provider to assess and intervene on behavioral health concerns. This partnership allows each provider to practice within their professional scope and to the top of their license and expertise while simultaneously advancing cross disciplinary knowledge, reducing care silos, enhancing cohesive care experiences for families, and building trust between families and their providers (12). Our model also considers and overcomes some of the structural and systems-related barriers previously identified by JM families including long wait times, difficulty with coordination of care, limited office hours, and lack of time off work, by using a single visit with coordinated scheduling (12). The co-located model also allows for direct in time communication between rheumatology and behavioral health providers thus providing ongoing real time education to providers around mutual disciplines.

Fawole and colleagues (2021) surveyed patients with pediatric rheumatologic illness, including JM, and their parents. Findings indicate a discrepancy between self-diagnosed and clinician-diagnosed anxiety (27% and 39% respectively) and depression (18% and 35% respectively). Patients reported that they were less comfortable than parents with mental health providers and barriers to treatment included fear that mental health providers did not understand rheumatologic diagnosis and fear around insurance coverage for mental health care (23). Our hope is that this this EBHC model also reduces mental health stigma and barriers to additional mental health care needs, by presenting preventative behavioral health care as a standard component of JM CoE visit.

The presence of a well-established JM CoE at our institution helped facilitate the development, funding, and implementation of this EBHC model. Other hospital systems may incorporate their JM patients in general rheumatology clinics thus making implementation of this EBHC model more challenging. Subdividing rheumatology clinics by diagnosis such as JM or lupus may allow for implementation of a similar model. Other possibilities include pursuing a generalized model that integrates EBHC across additional rheumatological subspecialty clinics or division wide. Successful implementation of the EBHC model requires not only inter-divisional/departmental alignment of priorities but also must include hospital leadership commitment and alignment with institutional strategic planning and funding efforts.

Institutional commitment may be enhanced by demonstrating that a preventative EBHC model can have significant impact on downstream patient outcomes and relieve some of the burden of the mental health crisis on the healthcare system. For example, quality improvement and research studies have illustrated many benefits of EBHC services including improved outcomes associated with shorter hospital admissions (24), adherence (25), decreased emergency department visits (26), reduced mental health symptoms (27), pain relief (28), improvement in quality of life, and equitable access to preventative medical and mental health care (29). Embedded behavioral health can also reduce emergency behavioral health spending by 19% (30) and offset medical costs by 20% (31).

In addition to stated clinical benefits of this model, it can also further research in mental health. The CARRA mental health workgroup identified research priorities around mental health in pediatric rheumatology diseases and four domains were thought to be important to be studied including identification of prevalence and incidence of mental health disorders, identification of mental health screening tools for this population, accuracy of mental health screens and or barriers around screening (32). We have demonstrated through our EBHC model that participation in mental health research is feasible and allows for much needed research data on these rare diseases (33).

In addition to the clear benefits of the EBHC model on patient outcomes and on institutional resource utilization, integration of behavioral healthcare is recommended in routine pediatric subspecialty care in other illness populations. For example, psychological care of children, adolescents, and young adults with diabetes is included in the International Society for Pediatric and Adolescent Diabetes Clinical Practice Consensus Guidelines (34). Similarly, the International Committee on Mental Health in Cystic Fibrosis recommend routine screening of anxiety and depression symptoms in youth with Cystic Fibrosis (35). A set of 15 standards for psychosocial care of children with cancer and their families (36) were also published in a special issue of Pediatric Blood and Cancer in 2015, which includes recommendations for routine assessment (37) intervention (38), among many others.

One of the key constraints of the EBHC model is identification of ongoing sustainable funding sources for the behavioral health provider’s salary. Due to low reimbursement rates of behavioral health services as well as nonbillable services, supplementation of salary and fringe benefits require institutional support. Salaries for licensed, independent behavioral health providers range significantly from psychiatrist on the high end with a mean annual wage of approximately $249,760 (39), to mental health counselors on the low end with a mean annual wage of $48,520 (40). Some EBHC programs may choose a funding model that does not require an independently licensed provider, such a psychology postdoctoral fellow whose annual stipend in 2023 is 56,880 (41), though supervision by a licensed psychologist is still required (and must be accounted for). An unpaid behavioral health trainee who is seeking clinical experience is also an option, though again, clinical supervision by a licensed behavioral health provider must be included in the budget. Another potential disadvantage of a trainee model is lack of clinical continuity for patients and families over time. Alternative creative funding models include deployment of underutilized or excess FTE in psychiatry or rheumatology (respective) divisions, leveraging other mental health therapists such as social workers, or fundraising through private donors is also possible. The presence of foundation support for JM through the Cure JM Foundation was crucial for the viability of our model and similar foundations for rare diseases can be leveraged across subspecialties.

Other barriers to establishing a EBHC model include a long-standing shortage of behavioral health providers (42–44) and that became amplified during the COVID-19 pandemic (45). In addition to workforce shortages, providing behavioral health professionals with necessary training and skill development in responding to the unique needs of youth with chronic illness remains a challenge. In the context of this workforce shortage, behavioral health needs have either been largely unmet, or have fallen to physicians to manage. Green et al. surveyed pediatric subspecialty fellows taking the 2020 American Board of Pediatrics subspecialty in-training examination about their attitudes toward mental health care and their perceived competence in addressing mental health needs of children with chronic diseases. The majority of subspecialty fellows felt (63%) they should be responsible to address emotional or mental health needs of children with chronic disease, but few felt competent or trained to do so. Pediatric professional societies are increasingly embracing mental health skills training. This is relevant because while 76% of pediatric rheumatology fellows (*n* = 82) were interested in providing mental health care, only 25% (95% confidence intervals 14.7–34.3) felt competent to do so exemplifying the need to train the future generations explicitly in behavioral care (46). One of the added benefits of our EBHC model is the capacity to provide interdisciplinary training to rheumatology or other pediatric subspecialty fellows. Consensus within pediatric professional societies and subspecialties boards on curriculum and required competencies for mental and behavioral health during subspecialty fellowship training is sorely needed.

While our model is in its nascent phases and we are refining aspects of this model over the next few years, it is clear that there is a tremendous current need to address behavioral health proactively for youth with chronic disease. Our paper describes a unique EBHC model that can be adapted to other rare disease populations across other health systems and at different institutions along with specific guidance related to implementation, structure and funding.

## Data availability statement

The original contributions presented in the study are included in the article/Supplementary materials, further inquiries can be directed to the corresponding author.

## Author contributions

SS, SE, SH, and JP contributed to conceptualization and implementation of the model. SS, JP, and SE wrote and edited the article. SH read, edited, and approved the final version of the article and was the clinical psychologist of the COE. All authors contributed to the article and approved the submitted version.

## Funding

SS and SH received salary support from Cure JM Foundation grants related to the JM CoE.

## Conflict of interest

The authors declare that the research was conducted in the absence of any commercial or financial relationships that could be construed as a potential conflict of interest.

## Publisher’s note

All claims expressed in this article are solely those of the authors and do not necessarily represent those of their affiliated organizations, or those of the publisher, the editors and the reviewers. Any product that may be evaluated in this article, or claim that may be made by its manufacturer, is not guaranteed or endorsed by the publisher.
