# Supplementary material for: Implementation of an embedded behavioral health care model in a pediatric rheumatology subspecialty juvenile myositis clinic
Source: Front Psychiatry. 2023 Aug 10;14:1192711. doi: 10.3389/fpsyt.2023.1192711 (PMC10447969; doi:10.3389/fpsyt.2023.1192711)
Supplement: Supplementary file 1 [file Data_Sheet_1.docx]

**Supplemental Table S1**

Embedded Behavioral Health Care Model in Juvenile Myositis Clinic – results for initial six month of pilot.

| Myositis Clinics | 8 |
| --- | --- |
| Unique Patients | 12 |
| Screenings Completed | 10 |
| Screened above clinical risk threshold | 2 |
| Referrals Provided (regardless of risk threshold on standardized measures) | 3 |

**Supplemental Figure S1**

Mental and Emotional Health Resources page developed by Cure JM for families with Juvenile Myositis


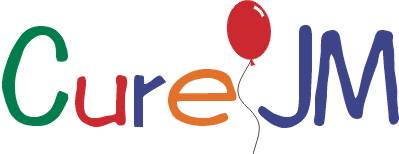


**MENTAL & EMOTIONAL HEALTH RESOURCES**

**from CURE JM for CURE JM FAMILIES**

- Facebook group: [Emotional & Mental Health Support Group](https://www.facebook.com/groups/curejmemotionalsupport)

This is a place to get support from other families, receive educational articles, information about events and mental health tips.

- Find [information about signs and symptoms of depression and anxiety](https://www.curejm.org/mental_health/mental-and-emotional-health-general-resources.php) and places to get help, nationally, including a downloadable flyer on Emotional Health and JM on the same page.
- Find more [Emotional & Mental Health information on these pages](https://www.curejm.org/mental_health/mental-and-emotional-health-home.php) on the Cure JM website.

Cure JM Foundation 19309 Winmeade Drive; Suite 204, Leesburg, VA 20176 www.curejm.com
